# Two-dimensional spin-gapless semiconductors: A mini-review

**DOI:** 10.3389/fchem.2022.996344

**Published:** 2022-08-25

**Authors:** Jianhua Wang, Dandan Wang

**Affiliations:** School of Physical Science and Technology, Southwest University, Chongqing, China

**Keywords:** two-dimensional material systems, spin-gapless materials, Dirac point, nodal line, spin transport properties

## Abstract

In the past decade, two-dimensional (2D) materials and spintronic materials have been rapidly developing in recent years. 2D spin-gapless semiconductors (SGSs) are a novel class of ferromagnetic 2D spintronic materials with possible high Curie temperature, 100% spin-polarization, possible one-dimensional or zero-dimensional topological signatures, and other exciting spin transport properties. In this mini-review, we summarize a series of ideal 2D SGSs in the last 3 years, including 2D oxalate-based metal-organic frameworks, 2D single-layer Fe_2_I_2,_ 2D Cr_2_X_3_ (X = S, Se, and Te) monolayer with the honeycomb kagome (HK) lattice, 2D CrGa_2_Se_4_ monolayer, 2D HK Mn–cyanogen lattice, 2D MnNF monolayer, and 2D Fe_4_N_2_ pentagon crystal. The mini-review also discusses the unique magnetic, electronic, topological, and spin-transport properties and the possible application of these 2D SGSs. The mini-review can be regarded as an improved understanding of the current state of 2D SGSs in recent 3 years.

## 1 Introduction

Due to their unique physical and chemical characteristics induced by low-dimensionality and electronic constraints, as well as their potential applications in spintronics, high-temperature ferromagnetic two-dimensional (2D) materials (Lee et al., 2010; Li and Yang, 2014; Wang et al., 2016a; Zhou et al., 2016; Ashton et al., 2017; Benmansour et al., 2017; Gong and Zhang, 2019; Kim et al., 2019; Zhou et al., 2019; Chen et al., 2020; Torelli et al., 2020; Xu et al., 2020; Zhang et al., 2021a; Tang et al., 2021; Miao and Sun, 2022) have attracted a great deal of attention in recent years. Nevertheless, the majority of prepared 2D materials that resemble graphene are not magnetic (Wang et al., 2012; Liu and Zhou, 2019), magnetic ordering has not been observed in the 2D material family for more than 10 years since the discovery of graphene (Hashimoto et al., 2004; Novoselov et al., 2004; Huang et al., 2017) in 2004. Recently, only some intriguing 2D magnetic materials, such as CrI_3_ (Huang et al., 2017), CrGeTe_3_ (Gong et al., 2017; Wang et al., 2018a), Fe_3_GeTe_2_ (Deng et al., 2018a; Fei et al., 2018), VSe_2_ (Bonilla et al., 2018) and CrTe_2_ (Sun et al., 2020a), have been experimentally realized. Furthermore, it should be noticed that, the 2D magnetic material is far from the actual spintronic application at room temperature due to the low Curie temperature *T*_*c*_ and low spin polarization. Thus, it is significant and urgent to develop ferromagnetic 2D materials with high spin-polarization and *T*_*c*_ via theory and experiment.

**FIGURE 1:**
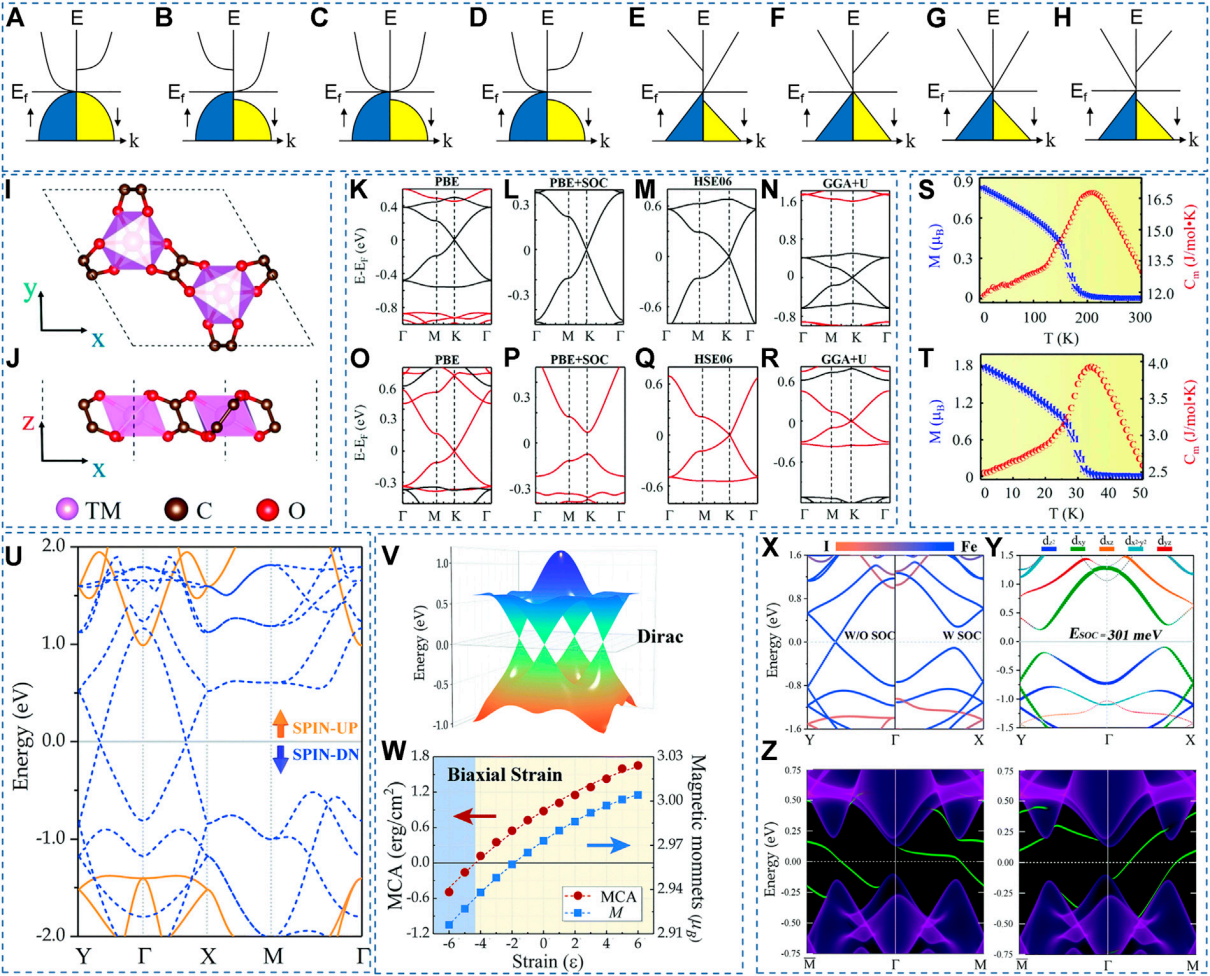
**(A–H)** Different SGSs. **(I–J)** Top and side views of the TM_2_(C_2_O_4_)_3_ structure. The calculated band structures (BSs) of Ni_2_(C_2_O_4_)_3_ **(K–N)** and Re_2_(C_2_O_4_)_3_ **(O–R)** with different methods. M and C_m_ of Ni_2_(C_2_O_4_)_3_ **(S)** and Re_2_(C_2_O_4_)_3_ **(T)** as a function of temperature. **(I–T)** Reproduced from (Xing et al., 2022) with permission from RSC publishing **(U)** BS of the Fe_2_I_2_ monolayer. **(V)** 3D plot of Dirac point **(W)** Magnetic anisotropy and magnetic moment of the Fe_2_I_2_ as a function of biaxial strain. **(X)** and **(Y)** atom-resolved BSs without and with SOC. **(Z)** Edge states of 2D Fe_2_I_2_ **(U–Z)** Reproduced from (Sun et al., 2020b) with permission from RSC publishing.

Among different types of 2D ferromagnetic materials, 2D spin-gapless semiconductors (SGSs) (Li et al., 2009; Zhang et al., 2015; Gao et al., 2016; Zhu and Li, 2016; Wang et al., 2017a; He et al., 2017; Lei et al., 2017; Wang, 2017; Deng et al., 2018b; Wang et al., 2018b; Wu et al., 2020a; Yang et al., 2020a; Wu et al., 2020b; Deng et al., 2020; Feng et al., 2020; Li et al., 2020; Nadeem et al., 2020; Rani et al., 2020; Wang et al., 2020; Yue et al., 2020; Şaşıoğlu et al., 2020; Feng et al., 2021; Phong and Nguyen, 2022) are ideal candidates for high-efficient spintronic devices. Wang (Wang, 2008) first proposed the concept of SGSs in 2008, and the SGSs can be viewed as a bridge to connect the magnetic semiconductors (Haas, 1970; Dietl, 2010; Sato et al., 2010) and half-metals (Wang et al., 2016b; Wang et al., 2017b; Wang et al., 2017c; Liu et al., 2017; Wang et al., 2018c; Han et al., 2019; Wang et al., 2019; Yang et al., 2020b; Tang et al., 2021; Yang et al., 2021). It is well known that the SGSs (Wang et al., 2018b) can host parabolic and linear dispersion between energy and momentum (see Figure 1A–H). Moreover, SGSs (Wang, 2017) can be categorized into four different types depending on the touching types of the valence band maximum (VBM) and the conduction band minimum (CBM) in both spin directions. We take the SGSs with parabolic dispersion as examples to introduce the above four types (see Figure 1A–D). In Figure 1A, one finds the CBM and VBM touch each other at the Fermi level (FL) in the spin-up (SU) channel, whereas a semiconducting gap appears in the spin-down (SD) channel. The VBM in the SD channel touches the FL. Figure 1B shows the semiconducting gaps in both spin channels. However, the VBM in the SU channel touches the CBM in the SD channel, forming an indirect zero-gap state. The case of Figure 1C is similar to that of Figure 1B. However, the CBM touches the FL in the SD channel. Figure 1D is the standard form of SGSs with parabolic dispersion: a zero-gap in the SU channel and a semiconducting gap in the SD channel. Similarly, the cases of SGSs with linear dispersion are listed in Figure 1E–H. Note that, for cases I, III and IV (see Figures 1A,C, D, E, G, F), depending on how the VBM and CBM touch each other, the zero-gap in one spin channel can be direct (VBM and CBM touch each other at the same *k* point) or indirect (they touch each other at different *k* points) (Wang et al., 2020).

SGSs may host the following advantages: 1) the excitation of electrons from the valence band to the conduction band requires only a tiny amount of energy. 2) the excited carriers (electrons and holes) can be fully spin-polarized (S-P) simultaneously. 3) one can use the Hall effect to separate the 100% S-P electrons and holes. 4) for the case II SGSs (See Figure 1B and Figure 1F), one can control the gate voltage to manipulate the SU and SD electrons and holes. 5) researchers proposed nodal point SGSs and nodal line SGSs in 2D and 3D materials, which can be excellent candidates for studying the relationship between topological and spintronics. For example, Dirac SGSs may induce low energy consumption and ultrafast transport because of their unique linear band dispersion. Hence, Dirac SGSs can cohost 100% spin-polarization and linear Dirac point at the FL.

Although there were several reviews on the research topic of SGSs, these articles (Wang, 2017; Wang et al., 2020; Yue et al., 2020) all focused on SGSs from 2008 to 2020. To our best knowledge, other researchers have not reviewed the recent advances in 2D SGSs from 2020 to 2022. From 2020 to 2022, a series of ideal 2D SGSs are proposed via first-principles calculations, and the related novel properties are also investigated. Therefore, for spintronics and topology, a mini-review of 2D SGSs seems necessary. It is noteworthy that Dirac SGSs and nodal line SGSs are new cross concepts in spintronics and topology. Although in almost all the reported 2D (2D) materials, the twofold degenerate nodal points in their band structures are misused as “Dirac points” due to a historical issue (Yang, 2016). The correct naming of these nodal points should be “Weyl”, and then each twofold degenerate point is described by the Weyl model in 2D. This review follows the common practice of using “Dirac point” SGSs in 2D materials.

In this review, we divided 2D SGSs into four classes: 2D SGSs with direct band crossing points at high-symmetry (H-S) points and along the H-S paths, 2D SGSs with indirect zero-gap states, and 2D SGSs with zero-gap nodal ring states. Note that this is the first time to review SGSs based on classification as mentioned above.

Herein, we will review the most recent investigations of 2D SGSs from 2020 to 2022. Section 2 introduces the proposed 2D SGSs with band crossing points at the H-S point. Section 3 introduces the proposed 2D SGSs with band crossing points along the H-S paths and their unique behaviors. Section 3 reviews 2D SGSs with indirect zero-gap states and their possible application. Section 4 introduces the case of 2D SGSs with zero-gap nodal ring states. Section 5 is the conclusion.

### 2 2D SGSs with band crossing points at H-S points

In 2022, Xing *et al.* (Xing et al., 2022) proposed a family of 2D oxalate-based metal-organic frameworks (MOFs) that possed the SGS characteristic. Figures 1I,J show the structure and reciprocal lattice of a 2D MOF TM_2_(C_2_O_4_)_3_ with a honeycomb-kagome (HK) lattice. Figure 1K–R show the electronic BSs of Ni_2_(C_2_O_4_)_3_ and Re_2_(C_2_O_4_)_3_ calculated by different methods along the Γ-M-K-Γ high symmetry paths. Without SOC, the valence band and conduction band in one spin channel touch the FL at the K point, and the other spin channel has a semiconducting band gap of 1 eV (see Figure 1K, O). Meanwhile, spin-gapless Dirac points with linear dispersion appear at the FL in one spin channel, which is beneficial for dissipationless spin transport. The influence of SOC on the Dirac point at the K H-S point is considered, and the results are shown in Figure 1L, P. One finds that the SOC triggers a band gap of about 7.6 meV in Ni_2_(C_2_O_4_)_3_ and 143 meV in Re_2_(C_2_O_4_)_3_, respectively. Compared with Ni_2_(C_2_O_4_)_3_, the SOC-induced gap of Re_2_(C_2_O_4_)_3_ is more significant than that of Ni_2_(C_2_O_4_)_3_ because the relative atomic mass of the Re atom is heavier than that of the Ni atom, and the Dirac point of Re_2_(C_2_O_4_)_3_ only contributes the *d* orbital of Re atom. Figure 1M, Q show the BSs calculated by the HSE06 method, and Figure 1N, R show the BSs calculated by the GGA + U method. One finds that the spin-gapless Dirac point is still maintained at the K point under both HSE06 and GGA + U methods.

With the PBE functional, the calculated Fermi velocity (*v*_*F*_) values (Xing et al., 2022) are up to 2.0 × 10^5^ m s^−1^ and 1.86 × 10^5^ m s^−1^ for Ni_2_(C_2_O_4_)_3_ and Re_2_(C_2_O_4_)_3_, respectively. When using the HSE06 functional, the obtained *v*_*F*_ values are relatively higher, up to 2.78 × 10^5^ m s^−1^ and 2.58 × 10^5^ m s^−1^ for Ni_2_(C_2_O_4_)_3_ and Re_2_(C_2_O_4_)_3_, respectively. As seen in Figure 1S, T, M and C_m_ exhibit a sudden change at a temperature of 208 K for Ni_2_(C_2_O_4_)_3_ and 34 K for Re_2_(C_2_O_4_)_3_, respectively. Note that the ultimate goals of spintronic or electronic devices in the future are ultra-fast transmission and extremely low energy consumption. The massless charge should ideally be fully S-P, and the (effective) mass of electrons or holes should be eliminated. Therefore, a class of magnetic materials called 2D SGSs with Dirac points at high symmetry points can be considered ideal for the use of next-generation spintronics (Wang et al., 2018b).

### 3 2D SGSs with band crossing points along the H-S paths

#### 3.1 Example 1: 2D single-layer Fe_2_I_2_

In 2020, Sun, Ma, and Kioussis (Sun et al., 2020b) proposed single-layer Fe_2_I_2_, with space group *P4/nmm* (nop. 129) and calculated lattice constants a = b = 3.81 Å, is a 2D SGS. The calculated BSs for single-layer Fe_2_I_2_ without SOC and with GGA + U are shown in Figure 1U. One finds that the SU bands show a semiconducting behavior, whereas the SD bands show a zero-gap behavior. Two gapless band crossing points appear at the FL in the SD channel. Unlike the gapless point at the H-S point in Ni_2_(C_2_O_4_)_3_ and Re_2_(C_2_O_4_)_3_, the gapless points in Fe_2_I_2_ are along the H-S paths. As shown in Figure 1U, the gapless points appear along the Y-Γ-X H-S paths. The 3D plot of these gapless points (named as Dirac points in Ref. (Sun et al., 2020b)) is shown in Figure 1V. The obtained *v*_*F*_ with the help of GGA + U and HSE06 is 4.66 × 10^5^ m s^−1^ and 6.39 × 10^5^ m s^−1^, respectively. As we all know, the massless Dirac fermions will lead to low effective masses and high carrier mobility. Further, as shown in Figure 1W, single-layer Fe_2_I_2_ undergoes a spin reorientation transition to an in-plane magnetization orientation beyond -4% compressive strain. As shown in Figure 1X, one finds that the SD bands arise from the Fe-d orbital, whereas the SU bands are from the I-p orbital. Hence, the Fe-d orbital contributes solely to the Dirac points at the FL. When SOC is added, significant band gaps (∼301 meV) appear along the Y-Γ-X H-S paths (see Figure 1Y) and a nonzero Chern number (|*C*| = 2). The edge states for the single-layer Fe_2_I_2_ are shown in Figure 1Z; one finds that two chiral topologically protected gapless edge states, which are consistent with the obtained |*C*| = 2. The SOC induces a physics nature transition from Driac SGS to quantum anomalous Hall (QAH) state in single-layer Fe_2_I_2_.

### 3.2 Example 2: 2D Cr_2_X_3_ monolayer with the HK lattice

**FIGURE 2:**
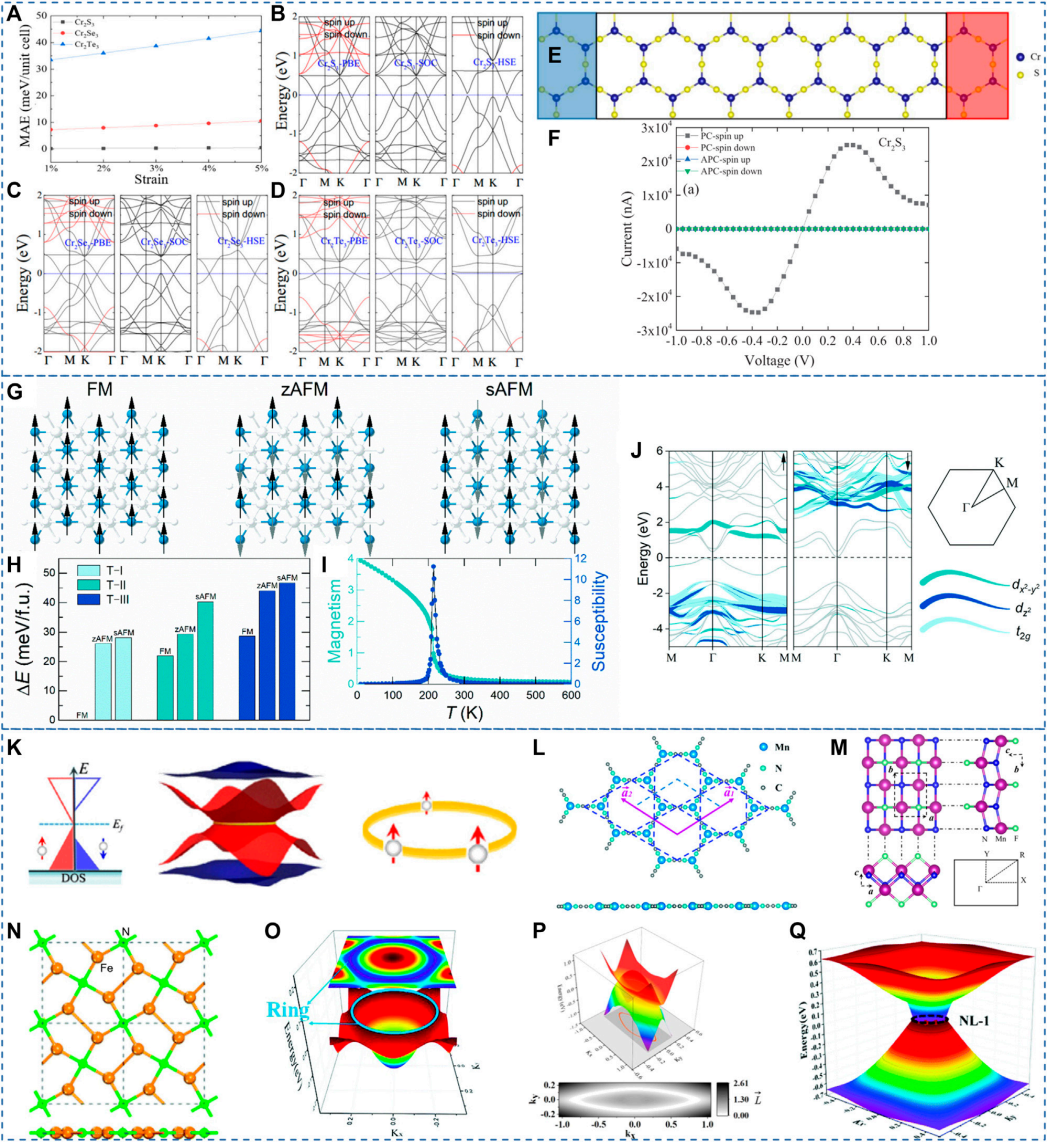
**(A)** The relationship between the MAE and strain. **(B–D)** BS of the Cr_2_X_3_ monolayers calculated with different methods. **(E)** The Cr_2_S_3_ device model. **(F)** The spin-resolved current-voltage curves for the PC and the APC of the device. **(A–F)** Reproduced from (Feng et al., 2021) with permission from AIP publishing. **(G)** Schematics for the FM and AFM states of the CrGa_2_Se_4_ monolayer. **(H)** Energy difference with respect to the ground state for T-I, T-II and T-III configurations. **(I)** The simulated Curie temperature **(J)** The calculated BSs by the HSE06 method. **(G–J)** Reproduced from (Chen et al., 2021) with permission from RSC publishing. **(K)** The schematic diagram of NRSGSs. Reproduced from (Zhang et al., 2020b) with permission from APS. **(L–N)** Structures of 2D HK Mn-cyanogen lattice, 2D MnNF monolayer, and 2D Fe_4_N_2_ pentagon crystal, respectively. **(O–Q)** 3D plot of the gapless NR states in 2D HK Mn–cyanogen lattice, 2D MnNF monolayer, and 2D Fe_4_N_2_ pentagon crystal, respectively. **(L–Q)** Reproduced from (Zhang et al., 2018; Hu et al., 2019; Zhang et al., 2021b) with permission from RSC and ACS publishing.

In 2021, Feng, Liu, and Gao (Feng et al., 2021) proposed the spin-gapless semiconducting states in 2D Cr_2_X_3_ monolayers (X = S, Se, and Te) via first-principle calculations. The estimated Curie temperatures for these three monolayers are about 420, 480, and 510 K, respectively. The S-P BSs and the calculated MAE for these three monolayers are collected in Figures 2B–D. One finds these three monolayers belong to 2D SGSs with zero-gap Dirac points along the H-S paths, i.e., K-Γ-M. As shown in Figure 2A one finds that the MAEs for these three monolayers increase with the increasing tensile strains from 1% to 5%. Unfortunately, the SGS behaviors in Cr_2_Te_3_ at the FL are destroyed within HSE06. For the Cr_2_S_3_ and Cr_2_Se_3_, the Dirac points along the K-Γ-M paths are still maintained within PBE and HSE06. The effect of SOC to the Dirac points is also examined by Feng, Liu, and Gao (Feng et al., 2021); they stated that the SOC effect is weak for the proposed monolayers.

Feng, Liu, and Gao (Feng et al., 2021) also studied the nonequilibrium spin transport properties of monolayer Cr_2_S_3_, and the device model is shown in Figure 2E. From Figure 2F, for the APC in both spin directions, one finds the values of spin-currents are extremely small. For the PC, one finds the spin-current of the PC-spin down can be neglected, whereas the spin-current of PC-spin up increased at first and then decreased with the increase of voltage form 0.0 V–1.0 V. The maximum value of spin current of PC-spin up appears at about+/-0.35 V. Hence, the device model in Figure 2E should host a perfect spin filtering effect (Chen et al., 2019; Zhang et al., 2020a; Han et al., 2022).

### 4 2D SGSs with indirect zero-gap states

In 2021, Chen *et al.* (Chen et al., 2021) predicted a 2D spin gapless ferromagnetic semiconductor of CrGa_2_Se_4_ monolayer with indirect zero-gap state. As shown in Figures 2G,H, one finds that the magnetic ground state is the FM state with a T-I configuration. It can be seen from Figure 2I that the Curie temperature of the CrGa_2_Se_4_ monolayer is about 220 K. Chen *et al.* calculated the BSs of the CrGa_2_Se_4_ monolayer with HSE06 functional. The results are collected in Figure 2J. At first glance, one finds that the CrGa_2_Se_4_ monolayer is a ferromagnetic semiconductor. The bands in SU and SD channels host semiconducting gaps of 0.36 eV and 1.36 eV, respectively. Interestingly, the lowest conduction band state in the SD channel touches the FL, and the highest valence band states in the SU channel touch the FL, forming an indirect zero-gap state. Hence, the CrGa_2_Se_4_ monolayer can also be seen as an SGS with an indirect spin-gapless semiconducting state.

We would like to point out that the indirect zero gap states occur because the two spin components at different *k* points accidentally have their extreme values at the FL. Therefore, in general, they are not protected from the symmetry of systems due to the indirect band touching. However, the SGSs with indirect band touching usually host bipolar magnetic behavior. That is, by changing the sign of the applied gate voltage, one can achieve the electrical manipulation of spin-polarization orientation in SGSs (with indirect band touching).

### 5 2D SGSs with zero-gap nodal ring states

Compared to the Dirac SGSs with single or multiple nodal point states, Zhang *et al.* (Zhang et al., 2018) proposed a new class of 2D SGSs with a gapless nodal ring (NR) in the momentum space and 100% spin polarization. That is, the SGSs, with a one-dimensional topological signature, have zero-gap band crossing points that form a line in the momentum space. Typically, they are named as NRSGSs. The schematic diagram of NRSGSs is shown in Figure 2K. One finds that the SU channel shows a zero-gap NR state in the momentum space and the SD channel shows a semiconducting state.

To this date, 2D HK Mn–cyanogen lattice (Zhang et al., 2018), 2D MnNF monolayer (Hu et al., 2019), and 2D Fe_4_N_2_ pentagon crystal (Zhang et al., 2021b) are proposed to be 2D NR SGSs. The structural model and the 3D plot of the gapless NR state in one spin channel are shown in Figure 2L–Q. We would like to point out that the gapless NR state in one spin channel may suffer sizable SOC-induced gaps. Hence, searching for NRSGSs with light elements to reduce the value of SOC-induced gaps.

## 6 Conclusion and remarks

In this mini-review, we introduced a series of ideal 2D SGSs, including 2D SGSs with band-crossing points at H-S points or along the H-S paths, 2D SGSs with S-P NR states, and 2D SGSs with indirect zero-gap states.

The Dirac SGSs with band-crossing points at H-S points or along the H-S paths show massless fermions around the FL, ideal dissipation-less properties, and 100% spin-polarization. Furthermore, the band crossing points may not isolate in the momentum space and form an NR in 2D SGSs. The NRSGSs will exhibit more intensive nonlinear electromagnetic responses than a single Dirac point. It should be noted that the 2D SGSs are hopped to host a high Curie temperature and a robust FM state at room temperature. Finally, a major challenge for 2D SGSs is that no 2D SGSs has been experimentally realized. The reason is that the 2D SGSs are monolayer materials, and they are hard to synthesize. Moreover, some monolayer materials are not stable in the ambient environment. Thus, new nanotechnology is needed for fabricating 2D monolayer SGSs.
